# Spatio-Temporal Regulation of IGFs in Enamel Development: Molecular Mechanisms From Ameloblast Polarity to Mineralization Homeostasis

**DOI:** 10.1155/sci/9665706

**Published:** 2025-05-13

**Authors:** Xue Zeng, Pengcheng He

**Affiliations:** ^1^Department of Pediatric Outpatient Nursing, West China Second University Hospital, Sichuan University, Chengdu, Sichuan, China; ^2^Key Laboratory of Birth Defects and Related Diseases of Women and Children, (Sichuan University) Ministry of Education, Chengdu, Sichuan, China; ^3^Department of Pediatric Dentistry, West China Second University Hospital, Sichuan University, Chengdu, Sichuan, China

**Keywords:** cell polarity, enamel formation, enamel mineralization, IGFs, PH homeostasis

## Abstract

The development of enamel relies on the precise regulation of ameloblast differentiation, enamel matrix secretion, and mineralization. The formation of enamel is crucial for the normal function of dental tissues, and promoting enamel remineralization is of significant importance for the treatment of dental caries. Understanding the underlying mechanisms of enamel development is essential for oral therapy and provides a bridge to tooth regeneration. Among various growth factors, the insulin-like growth factor (IGF) family, including IGF-1 and IGF-2, has been shown to play a key role in enamel formation by activating signaling pathways such as PI3K/AKT and MAPK. This review summarizes the role of the IGF family in tooth development and enamel formation and sheds light on key parts of the research for future treatment improvements.

## 1. Introduction

As the hardest mineralized tissue in the human body, enamel, with its highly ordered arrangement of hydroxyapatite crystals and unique protein matrix, confers excellent mechanical properties and carries resistance to teeth [1]. The formation of this special structure relies on the sophisticated spatial and temporal regulation of enamel-forming cell differentiation, enamel matrix secretion, and mineralization processes during tooth germ development. From plate formation in the embryonic stage to matrix degradation during enamel maturation, enamel-forming cells undergo complex biological events, including the establishment of polarity, the synthesis and secretion of matrix components such as amelogenin, and the fine regulation of microenvironmental ionic homeostasis [2–4]. Dysregulation of any part of this process may lead to enamel developmental defects, such as enamel mineralization insufficiency, enamel formation insufficiency, and other common clinical diseases, seriously affecting oral health and quality of life [5, 6].

In recent years, the role of the Insulin-like growth factors (IGFs) family in hard tissue development has attracted much attention. IGFs (mainly including IGF-1 and IGF-2) are widely involved in the regulation of cell proliferation, differentiation, and metabolism by binding to the specific tyrosine kinase receptor, IGF1R, and activating downstream signaling pathways such as PI3K/AKT and MAPK [7, 8]. In the field of dental embryo development, several studies have shown that IGFs play a key role in the spatial and temporal dimensions: in situ hybridization revealed that IGF-1 mRNA is expressed in the dental epithelium at the bud stage of mouse molar teeth and is significantly enriched in the enamel cell layer of the campanulate stage; whereas, IGF-2 was found to be highly expressed in the enamel knot in human embryos, suggesting that it may influence cusp morphogenesis by regulating enamel knot signaling centers [9, 10]. It is worth noting that not only do enamel cells act through the classical endocrine pathway, but they also produce IGF-binding proteins (IGFBPs) in an autocrine/paracrine manner, forming a local signaling regulatory network [11].

Although studies have initially revealed the expression characteristics of IGFs in enamel development, there are still many unanswered questions about their specific mechanism of action: firstly, it is not clear whether there is functional heterogeneity between IGF-1 and IGF-2 at different stages of enamel cell differentiation; secondly, it is still necessary to systematically analyze how the signaling of IGFs interacts with other key developmental pathways, such as FGF and BMP, to form an interactive regulatory network; In addition, whether the regulation of enamel matrix mineralization by IGFs involves microenvironmental regulatory mechanisms such as pH homeostasis and ion transport also needs to be explored in depth. Based on the recent research progress, this paper systematically reviews the multidimensional regulatory role of IGFs in enamel development, focuses on the molecular mechanism of IGFs on enamel cell fate determination, enamel protein synthesis, and the establishment of mineralized microenvironment, and looks forward to the translational potential of IGFs in regenerative medicine, to provide new theoretical perspectives for the study of enamel developmental biology.

## 2. Molecular Characterization of IGFs and Their Spatiotemporal Expression in Dental Embryo Development

### 2.1. IGFs and Their Receptor Systems

IGF family consists of IGF-1, IGF-2, their receptors (IGF1R, IGF2R), and six IGFBPs 1-6 [11]. Binding to IGF1R triggers receptor dimerization and autophosphorylation of *β*-subunit tyrosine residues, which activates the classical downstream pathways: (i) PI3K/AKT pathway: promotes cell survival and metabolism regulation by phosphorylating AKT, which plays a central role in enamel-forming cells' energy supply; and (ii) MAPK/ERK pathway: regulates the expression of cell cycle proteins (e.g., cyclin D1) to drive enamel-forming cells' proliferation [12, 13]. Notably, the functional divergence between IGF-1 and IGF-2 may stem from differences in their receptor binding modes. Although both rely primarily on the IGF1R for signaling, IGF-2 has more than 100-fold higher affinity for the IGF2R (i.e., cation-independent mannose-6-phosphate receptor) than IGF-1 [14]. This property suggests that at specific stages of tooth germ development (e.g., enamel degradation), IGF-2 may remove locally excessive IGFs through IGF2R-mediated endocytosis, forming a “self-braking” mechanism for dynamic homeostasis. In addition, glycosylation modifications of IGFBPs may confer spatiotemporal-specific regulatory capabilities: mass spectrometry analysis showed that IGFBP5 secreted by enamel-forming cells exhibits a unique O-GlcNAc modification during the bell-shaped phase, and this posttranslational modification may regulate the bioavailability of IGF-1 by altering its ability to bind to the extracellular matrix. This hypothesis can be tested in organoid models by constructing glycosylation site mutants [12].

### 2.2. Dynamic Expression Patterns in Tooth Germ Development

By double fluorescence staining technique, the researchers revealed the spatiotemporal specificity of IGFs in dental embryo development: bud stage to cap stage: IGF-1 and HSP70 expression in mouse molar dental epithelium was higher than that in the mesenchyme, suggesting that they dominate epithelial-mesenchymal interactions; bell stage: the peak expression of IGF-1 in enamel precursor cells were synchronized with the degradation of enamel nodes, while the expression of HSP70 in this stage less coincided with the disintegration of primary enamel nodules; IGF-2 was highly expressed mainly in enamel-forming vessels, while PTEN was seen to be mild to moderately expressed in papillae and some enamel organs. IGF-1R expression was ubiquitous and showed strong intensity throughout the enamel organs. In contrast, IGF-2R expression has a rather irregular pattern in enamel organs and dental papillae [15–17].

When the tooth tissue is affected by trauma or caries, IGFs have been proved to be involved in the formation of tertiary dentin and the defensive activities of the tooth, which can promote the formation and recruitment of odontoblast-like cells and the formation of dental mineralized tissue [9]. This whole process is closely related to the ability of odontoblasts and ameloblasts derived from cranial nerve crest to maintain the persistence of embryonic programs. d'Aquino et al. [18] found that neural crest-derived cells (DFC) co-express early neural progenitor cell markers and vascular endothelial growth factor receptors, including Brn3a and flk-1, and retain embryonic markers and transcription factors, such as Oct-4, nanog, TRA1-60, and TRA-1-80-1. And can form neurons, osteoblasts, adipocytes, and other cell types after being stimulated by relevant differentiation signals, which is also an important basis for odontoblasts and ameloblasts to participate in dental cell events at birth or adulthood.

The spatial and temporal expression profiles of IGFs revealed that they may be involved in the regulation of “dual signaling centers” in the formation of dental embryonic patterns: high expression of IGF-2 in early enamel junctions for the early proliferation and differentiation of primary progenitor cells, and the expression of IGF-1 in the late enamel cell layer overlapping with HSP70 signaling. We hypothesize that IGF-2 may initiate the “initiation wave” of apical morphogenesis by activating the primary signaling of enamel nodule cells, while IGF-1 ensures the regular arrangement of the enamel column structure by maintaining the homeostasis of the enamel precursor cell pool in the subsequent stages.

## 3. Regulation of Enamel Cell Differentiation by IGFs

### 3.1. Promote the Proliferation and Differentiation of Enamel-Forming Cells and the Establishment of Cell Polarity

Scholars found that GHR and IGF1 proteins were detected in both incisors and molars of rat embryos and were highly expressed in dental epithelial cells, suggesting that the GH/IGF axis plays a regulatory role in their value-added differentiation process and that treatment with GH in vitro promotes the further expression of IGF1 proteins in the epithelial cells and promotes epithelial cell proliferation and differentiation [19–21]. From the bud stage to the capitulum stage, IGF-1 was expressed in the dental plate and inner enamel cell layer, and the epithelial cells proliferated rapidly in this stage, and with the entry into the campanulate stage, the expression of IGF-1 declined, and was gradually confined to the dental plate area, while stellate reticulation cells appeared, which signaled the formation of the stellate layer. In the late campanulate stage, IGF-1 is only expressed in the apical region and cervical ring of the future teeth [22, 23], while IGF-2 is expressed in the epithelial tissues throughout the process, and forms a negative feedback mechanism with PTEN to regulate the proliferation and differentiation of enamel cells [24, 25].

The polarity-building process of enamel cells, in which the nucleus is located away from the basement membrane and the organelles are abundant and located in the near-basement membrane direction, is extremely important for the differentiation and maturation of enamel cells, as well as for their functional perfection. The polarity establishment process of enamel cells is mainly related to RhoA, Rac1, and CDC42 of the Rho family of proteins, which regulate the polymerization of the actin microfilament backbone through the rho-associated coiled-coil containing kinases (ROCK) signaling pathway, thus affecting the polarity and morphology of the cells [26]; Rac1 expression pattern during rat molar development is similar to that of RhoA, with strong positive expression during dental embryo morphogenesis and enamel cell differentiation, and enhanced expression of Rac1 in polarized enamel cells, which is mainly expressed in the cytoplasm away from the side of the basement membrane with a punctate distribution, but the specific pathway mechanism needs to be further investigated; CDC42 showed a highly dynamic spatiotemporal expression pattern in developing enamel-forming organelles, and was strongly expressed in the outer enamel epithelium, the stellate reticular layer and the middle layer. It has been found that mice with epithelial cell-specific knockout of CDC42 have thinner enamel, chalky incisors, easily abraded molar teeth after eruption, reduced microhardness and shear resistance compared with the normal group, and disorganized enamel column structure as shown by scanning electron microscopy. CDC42 and Rac1 can interact with laminin-10/11-integrin *α*6*β*4 (laminin- 10/11-integrin *α*6*β*4), which regulates cell polarity and affects the size and shape of the dental embryo through the phosphatidylinositol 3-kinase-CDC42/Rac pathway [27, 28]. Some studies have shown that IGF-1 can be involved in the RhoA/ROCK signaling pathway, activating cancer cells to promote cell scattering and invasion [29], and can also activate the up-regulation of P13K through the IGF-1/IGF-1R-PI3K/Akt pathway [30], which can indirectly affect the process of enamel cell polarization, and the specific mechanisms need to be further investigated.

### 3.2. Regulation of Enamel-Specific Gene Expression

Some researchers placed the first molar teeth of mice in Petri dishes containing IGF-1 and IGF-2 and reverse transcribed the extracts at 6, 12, and 18 days, respectively, and found that the concentrations of enamelogenic and enamel-forming proteins, mrana, were increased, suggesting that IGF-1 and IGF-2 can promote the synthesis of enamel-associated proteins [31]; at the same time, it was found that activation of IGF1R recruited the histone acetyltransferase p300 to the Runx2 promoter region, which increased the level of H3K27ac modification and in turn upregulated the transcription of the gene coding for the amelogenin protein. The spatial proximity of the Runx2 binding site to the topologically associated domain (TAD), where the amelogenin gene is located was increased after IGF-1 stimulation [32–34]. This suggests that IGFs may regulate the amelogenin gene cluster through three-dimensional genome remodeling, altering the chromatin spatial conformation and collaborating with multiple distal regulatory elements to drive amelogenin expression.

## 4. Dual Role of IGFFs in Enamel Matrix Mineralization

### 4.1. Promote Calcium and Phosphorus Deposition

It has been found that IGFs regulate mineralization kinetics through multiple dimensions: (i) Ion transport: IGF-1 promotes the translocation of TRP channels located in the intracellular pool to the plasma membrane, thereby enabling Ca^2+^ inward flow, and we hypothesize that this mechanism promotes calcium ion deposition in enamel and regulates cellular activity [35, 36]; and (ii) matrix regulation: matrix metalloproteinases are involved in the modification, degradation, and clearance of enamel matrix and enamel progenitor proteins, which is extremely important to the formation of enamel, studies have shown that IGF-1 and IGF-2 can activate matrix metalloproteinases through PI3K/AKT pathway, and we speculate that this mechanism can regulate enamel formation [37, 38]. The regulation of mineralization kinetics by IGFs may follow the “ion-substrate double-lock model”: on the one hand, IGF-1 and IGF-2 activate enamel metalloproteinases through TRP, and on the other hand, it protects the enamel matrix scaffold by regulating MMP-20, which synergistically ensures the deposition of hydroxyapatite along specific orientations.

### 4.2. Regulation of pH Homeostasis

**Figure 1:**
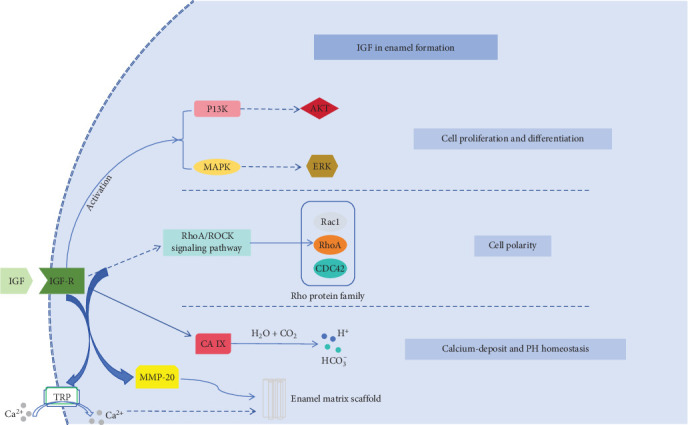
The postevent of IGF1 and IGF1R binding in cells.

It has been found that IGF-1 can regulate intracellular PH homeostasis and inhibit its acid efflux under hypoxic conditions, which is mainly achieved through the maintained mitochondria-derived PI-3 kinase-dependent pathway downstream of ROS [39]. Meanwhile, in the acidosis experiment in rats, the acidosis indicator HCO_3_- of the experimental group treated using IGF-1 changed from 20.4 ± 0.4 mM to 22.0 ± 0.5 mM, suggesting that it has a positive effect on the regulation of intracellular PH homeostasis [40]. Carbonic anhydrase (CA) can regulate PH homeostasis by generating hydrogen ions and bicarbonate ions. CA IX has four structural domains, which are proteoglycan-like, catalytic, transmembrane, and intracellular structural domains, among which the intracellular structural domain can participate in the negative feedback regulation of aerobic glycolysis by the PI3K/AKT pathway, and we hypothesized that IGF-1 may affect cellular PH homeostasis through this pathway [41] (Figure 1).

## 5. Interaction of IGFs With Other Signaling Pathways

There are also interactions of IGFs with other signaling pathways in the regulation of cellular activity. Inhibition of the FGF pathway can indirectly promote cell differentiation, which is balanced by IGF-mediated self-renewal. At the same time, the FGF signaling pathway promotes the secretion and production of IGF-2 by fibroblast-like cells, which inhibits cell proliferation [42]. IGFBP-3 forms a ternary complex with IGF-,1 prolonging its half-life in the circulatory system, enhancing its promotion of proliferation and differentiation [43], and also inhibiting the smad binding element (SBE) induced by BMP-2 signaling reporter activity, reducing the effect of BMP-2 on ALP activity and mineral nodule formation [44]. YAP helps to maintain stem cell properties as well as influencing organ development, especially organ size has an important role, and it was found that the expression of YAP is regulated by IGF-1, and at the same time, the upregulation of YAP also promotes the upregulation of IGF-1, forming a positive feedback regulatory mechanism [45]. Shh has been shown to promote cell proliferation and differentiation, and at the same time affect Akt phosphorylation through its effector Smo. It was found that the combination of Shh and IGF-1 had a superimposed effect, enhancing Akt and MAPK/ERK (p42/44) phosphorylation, and that IGF-1R integrates with Smo at the receptor level, and can respond to both Shh and IGF-1 [46, 47].

Wnt and Notch are highly conserved basic signal pathways among species, which play a key role in embryonic development, self-renewal of tissue stem cells, cell-specific differentiation, and other important life processes. These two pathways are also widely involved in the development of ameloblasts. When *β*-catenin, the core molecule of Wnt signaling pathway in epithelial cells, is knocked out, the development of tooth germ will be stopped in the early bud stage, and a large amount of accumulated *β*-catenin will continue to activate Wnt/*β*-catenin signaling pathway, leading to the formation of new glazes, which will eventually lead to redundant teeth and ectopic teeth [48, 49]. Fan et al. [50] found that the enamel mineralization of mouse incisors was delayed after activating *β*-catenin in ameloblasts derived from epithelial cells, which eventually led to insufficient mineralization of incisors. Notch signaling pathway component exists in Enamel-forming ameloblasts and the underlying stratum intermedium (SI) [51]. It is found that when the Notch signaling pathway is inhibited, it will lead to the defect of interaction between ameloblasts and Si cell layer and eventually lead to abnormal enamel development, meanwhile, it will also lead to the down-regulation of desmosome-specific proteins, such as PERP and desmosome protein, which will affect the attachment of ameloblasts and the integrity of enamel. At the same time, some researchers [52] found that when Notch pathway was inhibited, it not only inhibited the expression of neural spinal cell core factor of dental pulp stem cells, but also led to the silence of Wnt signal and the obvious decrease of stem cell potential of DPSCs. The activation of Wnt signaling pathway can not only enhance its stem cell potential, but also enhance the function of Notch signaling pathway and promote the expression of related molecules, which shows that the cross-linking effect of Notch/Wnt cross-signaling in tooth development is extremely important to the fate of cells. Jing et al. [53] found that the inactivation of IGF signal led to the decrease of transitional amplifying cells (TACs) and the damage of Wnt signal in incisor mesenchymal cells. At the same time, mesenchymal stem cells and dental follicle secreted IGF 2 ligand, and the TACS was regulated by IGF-Wnt signal cascade. After activating PI3K/AKT pathway, IGF can inhibit the activity of GSK-3*β*, thus indirectly stabilizing *β*-catenin and promoting Wnt signal pathway [54], and can also activate autocrine or paracrine Wnt signal by promoting the secretion of Wnt ligand [55]. Izrailit et al. [56] found that MAPK-ERK signaling pathway can play an important role in the development of breast cancer through Jagged 1 as a regulator of Notch signaling pathways. These studies show that IGF is involved in the regulation of Wnt and Notch signaling pathway indirectly or directly, thus affecting the proliferation and differentiation of mesenchymal cells into ameloblasts and the formation of enamel (Table 1).

## 6. Conclusion

The IGF family is widely involved in the proliferation, differentiation, and metabolic regulation of enamel-forming cells. Previous studies confirmed the spatiotemporal expression of the IGF axis in enamel development, which aroused research interest in the role of IGFs in tooth development. Further studies revealed that the IGF axis is involved in enamel formation and has important roles in cell differentiation and proliferation, polarity establishment, promotion of calcium and phosphorus deposition, and regulation of cellular homeostasis. In addition, the Ras/Raf-1/MAPK and PI3K/AKT/mTOR pathways were found to be activated by the IGF axis in this biological process. However, in-depth exploration of signaling pathway interactions, species differences, and therapeutic safety is still needed, and manually controlled release of the IGF axis mimicking biological processes has not yet been investigated. The afore-mentioned gaps also remind us of potential obstacles in the future application of IGFs. Looking ahead, further research on growth factors at the genomic level of biologically relevant cell types and potential therapeutic applications will be crucial, and enamel regeneration strategies targeting IGFs are expected to be a hot spot for future research.

## Figures and Tables

**Table 1:** Interaction of IGFs with other pathways and related mechanisms.

Pathway	Effect	Mechanism	Reference
PI3K/AKT	Activation	Cell survival, proliferation, and metabolism	[12, 13]
MAPK/ERK	Activation	Cell survival, proliferation, and differentiation	[12, 13]
CDC42/Rac	Promotion	Cell polarity, size, and shape of the dental embryo	[27, 28]
RhoA/ROCK	Promotion	Cell scattering and invasion	[29]
TRP channel	Transfer to plasma membrane	Promote calcium and phosphorus deposition	[35, 36]
CA IX	Negative feedback regulation	Regulation of pH homeostasis	[41]
FGF	Inhibition	Cell survival and differentiation	[42]
BMP/Smad	Inhibition	ALP activity and mineral nodule formation	[44]
YAP	Positive feedback regulation	Maintain stem cell properties and organ size	[45]
Shh	Promotion	Cell proliferation and differentiation	[46, 47]
Wnt	Promotion	Embryo development, self-renewal of tissue stem cells, and cell-specific differentiation	[48–50, 54, 55]
Notch	Promotion	Embryo development, self-renewal of tissue stem cells, and cell-specific differentiation	[51, 52, 56]

## Data Availability

The data sharing is not applicable to this article as no new data were created or analyzed in this study.
